# Microalbuminuria in Type-2 Diabetes Mellitus; the tip of iceberg of diabetic complications

**DOI:** 10.12669/pjms.333.12537

**Published:** 2017

**Authors:** Tauseef Ahmad, Imran Ulhaq, Minaz Mawani, Najmul Islam

**Affiliations:** 1 Dr. Tauseef Ahmad, FCPS. Endocrinology Section, Department of Medicine, Aga Khan University Hospital, Karachi, Pakistan; 2 Dr. Imran Ulhaq, FCPS. Endocrinology Section, Department of Medicine, Aga Khan University Hospital, Karachi, Pakistan; 3 Minaz Mawani, M.Sc. (Epidemiology and Biostatistics). Endocrinology Section, Department of Medicine, Aga Khan University Hospital, Karachi, Pakistan; 4 Dr. Najmul Islam, FRCP. Endocrinology Section, Department of Medicine, Aga Khan University Hospital, Karachi, Pakistan

**Keywords:** Complications, Hypertension, Microalbuminuria, Type-2 diabetes

## Abstract

**Objective:**

To determine the prevalence of microalbuminuria and its association with hypertension and other diabetic complications among Type-2 diabetic patients attending at Aga Khan University Hospital Karachi.

**Methods:**

1280 Type-2 diabetes patients who visited the outpatient department of Aga Khan University Hospital from September 2014 to August 2016 were included in the study. Microalbuminuria was diagnosed if spot urinary microalbumin excretion was confirmed to be more than 20mg/l. Hypertension was diagnosed if BP >140/90 or already on antihypertensive medications. Other demographic, clinical and laboratory data were also recorded.

**Results:**

Microalbuminuria was diagnosed in 404(31.56%) patients and among these albuminuric patients 335(82.9%) had hypertension. They were also dyslipidemic, having raised triglyceride levels, lower HDL levels, with more prevalence of background diabetic retinopathy and peripheral neuropathy. They also showed higher HbA1C levels and longer duration of diabetes.

**Conclusion:**

The prevalence of the microalbuminuria in our patients with Type-2 diabetes is 31.56% and is not only an early sign of diabetic nephropathy but also a host of other diabetic complications and should be dealt early with strict control of their hyperglycemia and hypertension to help prevent the future complications.

## INTRODUCTION

Diabetic nephropathy is one of the most common and feared complication of diabetes mellitus. As the burden of diabetes is increasing worldwide, more and more patients with diabetic nephropathy are surfacing. It has also been associated with increased morbidity and mortality and it is one of the most common cause of end stage renal disease(ESRD) and initiation of renal replacement therapy.^1^,^2^

Worldwide the burden of dialysis patients has doubled from 12.7 million in 1990-1991 to 23.6 million in 1998-1999^3^ and it is still increasing. Even thoughboth Type-1 and Type-2 diabetes mellitus(DM) can progress to ESRD but Type-2 are more commonly seen because of higher prevalence.^4^ As WHO has forecasted an increase in prevalence of diabetes around the globe with particular importance of increasing obesity,^5^ we expect exceptional increase in diabetic nephropathy worldwide.

From the early 80s, it was established that microalbuminuria is a good marker of subsequent proteinuria and chronic renal failure in both insulin and non-insulin dependent DM patients,^6^ but in Type-2 DM patient it has increased mortality rate mainly from cardiovascular diseases.^1^ It was also observed that these patients had concomitant hypertension as well when their microalbuminuria is diagnosed.^7^ Studies have shown that microalbuminuria is not only an independent risk factor for cardiovascular diseases in hypertensive and diabetic patients but also for general population^8^ and it is also an important tool for predicting the mortality and morbidity in patients with cardiovascular and peripheral vascular diseases.^9^ American Diabetes Association (ADA) has already included the screening of microalbuminuria in standards of medical care in diabetes.^10^

As little work has been done in this area in Pakistan and the economic cost of this disease is huge we need constant work in this regard so that we could find out possible preventive strategies. Our study reports the prevalence of microalbuminuria and its association with hypertension and other diabetic complications in adult patients with Type-2 DM.

## METHODS

All patients with Type-2 DM who visited outpatient department of Aga Khan University Hospital from September 2014 to August 2016 were included in our study. Type-2 DM was diagnosed by ADA criteria. Patients who had active urinary tract infections, menstruating women, hematuria due to any cause or had previous urinary tract surgery or malignancy were excluded from the study.

In all these patients, body mass index (BMI) measurements, examination for retinopathy, neuropathy and peripheral vascular disease were done and recorded. BP was recorded from arm by digital sphygmomanometer while at rest for 5 min. Patients having BP >140/90 or already on antihypertensive medications were labeled as hypertensive. Patient’s demographics and other laboratory parameters were recorded. Microalbuminuria was recorded by checking the spot urine microalbumin levels.

Other diabetic complications like peripheral neuropathy and retinopathy were also checked. Peripheral neuropathy was checked with monofilament and tuning fork test while retinopathy was observed by direct ophthalmoscopy. Peripheral vascular disease is checked by checking the peripheral pulsations. Previous history of ischemic heart disease and depression was also noted.

Statistical analysis was done by using SPSS version 19. Means with standard deviation (SD) were reported for all normally distributed quantitative variables such as Age, years with diabetes, HbA1c (glycated hemoglobin), triglycerides and HDL (high-density lipoprotein). Frequencies with percentages were reported for all categorical variables such as gender, comorbid conditions and medications. Prevalence of micro albuminuria was reported in Type-2 diabetes patients. Independent sample t-test was used to compare quantitative variables across the categories of microalbuminuria vs. no microalbuminuria. Chi square test was used to compare qualitative variables across categories. Logistic regression was done to identify association of microalbuminuria with hypertension amongst Type-2 diabetic patients. Adjusted odds ratio with 95% confidence intervals was reported. A P-value of <0.05 was considered as statistically significant.

## RESULTS

**Fig.1:**
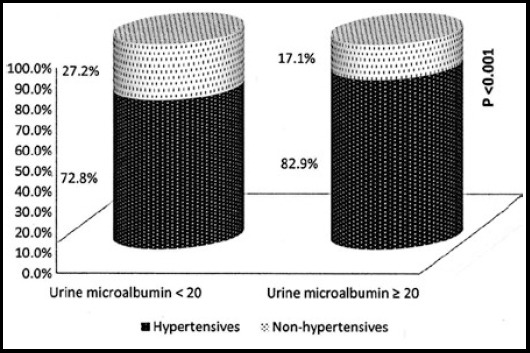
Distribution of hypertension between patients with microalbuminuria and those with no microalbuminuria.

A total of 1280 patients visited outpatient diabetes clinics from September 2014 to August 2016 with mean age of 54.9 ± 11.6 years. About 76% of the patients were hypertensive. Microalbuminuria was diagnosed in 404(31.56%) patients out of which 335(82.9%) had hypertension (Fig.1). Most of the patients (78.4%) were obese with a BMI of 25 or above. A small number of participants reported to be current or ex-smoker (16%). On an average, the study participants had been diagnosed with diabetes for about 9.7 ± 7.8 years with around 63.3% of the participants having the disease for more than 5 years. Dyslipidemia and coronary artery disease were diagnosed in 59.6% and 10.9% respectively. Mean HbA1c level was 7.65 ± 1.64. About 63.8% of the participants were on antihypertensive medications (22.3% on ACE, 30.3% on ARBs, 25.2% on beta blockers and 17.1% were on calcium channel blockers). In addition, 64.8% of the participants were on lipid lowering agents, 49.7% were on anti-platelets, 10.2% on pre-gablin, 79.3% on metformin, 41.3% on sulfonylurea, 59.6% on DPP4 inhibitors and 39.5% were on insulin.

Comparison of demographic and clinical factors across the categories of urine micro albumin levels are presented in Table-I. Male preponderance was observed in microalbuminuria with 55.2% of those having microalbuminuria level ≥ 20, being men. A higher percentage of the patients with microalbuminuria had hypertension, dyslipidemia, background diabetic retinopathy, peripheral neuropathy and raised triglyceride levels whereas these patients had lower mean HDL levels (41.3 ± 10.0 vs. 43.8 ± 11.7, P= 0.001). On an average, patients with microalbuminuria had higher HbA1c levels and longer diabetes duration. Age, BMI, smoking status, CAD, LDL, total cholesterol, SGPT, peripheral vascular disease, use of antiplatelet agents and oral hypoglycemic medications were not significantly different across the categories of urine microalbumin levels.

**Table-I:** Comparison of demographic and clinical characteristics of 1280 participants presenting to diabetes outpatient clinics.

*Characteristics*	*Overall (n=1280)*	*Urine Micro albumin <20 (n=876)*	*Urine Micro albumin ≥ 20 (n=404)*	*P-values*
Age (years), mean ± SD	54.9 ± 11.6	54.8 ± 11.4	55.1 ± 12.0	0.66
***Gender***				
Male	638(49.8)	415(47.4)	223(55.2)	0.009
Female	642(50.2)	461(52.6)	181(44.8)	
***Hypertension***				
Present	973(76)	638(72.8)	335(82.9)	<0.001
Absent	307(24)	238(27.2)	69(17.1)	
Years with diabetes, mean ± SD	9.7 ± 7.8	9.3 ± 7.5	10.5 ± 8.2	0.01
***HbA1c (%)***				
≤7.0	526(41.1)	397(46.2)	129(32.6)	<0.001
7.1-8.0	339(26.5)	225(26.2)	114(28.8)	
8.1 and above	391(30.5)	238(27.7)	153(38.6)	
***Dyslipidemia***				
Present	763(59.6)	494(56.4)	269(66.6)	0.001
Absent	517(40.4)	382(43.6)	135(33.4)	
***Triglycerides (mg/dl)***				
Normal (<150)	701(54.8)	502(64.8)	199(55.4)	0.003
High (150 and above)	433(33.8)	273(35.2)	160(44.6)	
***HDL (mg/dl)***				
Low(≤49 in female, ≤ 39 in male)	564(58.2)	376(56.3)	188(62.7)	0.06
Normal (50 and above for females, 40 and above for males)	404(41.7)	292(43.7)	112(37.3)	
***LDL***					
<100	884(72.6)	603(72.0)	281(73.9)	0.49
100 and above	333(27.3)	234(28.0)	99(26.1)	
***Background Diabetic Retinopathy***				
Present	58(4.5)	30(4.6)	28(9.7)	0.002
Absent	886(69.2)	626(95.4)	260(90.3)	
***Peripheral neuropathy***				
Present	208(16.3)	120(14.7)	88(24.2)	<0.001
Absent	972(75.9)	696(85.3)	276(75.8)	
***Use of Hypoglycemic agents***				
Only Insulin	93(7.3)	61(7.0)	32(7.9)	<0.001
Only OHA	758(59.2)	558(63.7)	200(49.5)	
Insulin and OHA	413(32.3)	246(28.1)	167(41.3)	
Neither insulin nor OHA	16(1.3)	11(1.3)	5(1.2)	
***Use of Statins***				
Yes	830(64.8)	550(62.8)	280(69.3)	0.02
No	450(35.2)	326(37.2)	124(30.7)	

SD: standard deviation; HbA1c: glycated hemoglobin; HDL: high density lipoprotein; OHA: oral hypoglycemic agents

Factors associated with urine micro albuminuria were examined using logistic regression models. On univariate level, hypertension, male gender, dyslipidemia, HbA1c levels, higher triglyceride levels, low HDL, use of hypoglycemic, lipid lowering drugs and pre-gablin was significantly associated with microalbuminuria. In the multivariate model adjusted for gender, HbA1c and dyslipidemia, we found that being hypertensive as compared to non-hypertensive was associated with 78% higher odds of microalbuminuria (aOR1.78, 95% CI: 1.30 to 2.43).Table-II. There was no confounding and interaction found in the model.

**Table-II:** Factors associated with Microalbuminuria in 1280 patients presenting to diabetes outpatient clinics.

*Variables*	*Unadjusted OR(95% CI)*	*Adjusted OR(95% CI)*	*P-values*
***HTN***			
Yes	1.81(1.34 to 2.44)	1.78(1.30 to 2.43)	<0.001
No	1	1	
***Gender***				
Male	1.36(1.08 to 1.73)	1.43(1.11 to 1.82)	0.004
Female	1	1	
HbA1c (%)	1.20(1.12 to1.29)	1.22(1.14 to 1.32)	<0.001
***Dyslipidemia***			
Yes	1.54(1.20 to 1.97)	1.52(1.17 to 1.97)	0.001
No	1	1	

OR: odds ratio; HbA1c: glycated hemoglobin; HTN: hypertension

## DISCUSSION

The prevalence of microalbuminuria in our population is 31.56% and it is comparable with the famous United Kingdom Prospective diabetes study that showed the prevalence of nephropathy as 30.8%.^11^ In Asian countries the prevalence of microalbuminuria ranges from 14.2% in Iran to 36.3% in India.^3^ MAP study reported the highest (56.5%) prevalence of microalbuminuria in Korea while the lowest (24.2%) in Pakistan.^12^ This huge variation in prevalence may be as a result of their life style, education level and ethnic variability.

Our study showed a significant larger male population in microalbuminuric group as compared to females and it is also observed in previous studies both in Pakistan and abroad.^13^ The cause of this phenomenon should be investigated in further studies. Our study also fails to demonstrate any difference between these two groups based on their BMI and it is in accordance of previous study^14^ and it is also due to the fact that majority of our study subjects in both groups were either overweight or obese.

Microalbuminuria is not only a risk factor for end stage renal failure in diabetes but also an important marker of mortality in diabetic populations.^1^ ADA has stressed for early detection of microalbuminuria in diabetes patients as early treatment of microalbuminuria retard the progression of diabetic nephropathy.^6^ There are multiple risk factors for microalbuminuria and studies have shown poor glycemic control and increased duration of diabetes, male gender and increased creatinine as important risk factors.^13^

Hypertension is not only a risk factor but also has very strong relationship with microalbuminuria^7^,^11^ and the same is evident in our study as well. Our study showed that significantly more patients with microalbuminuria had hypertension and majority 674(69.2%) were on angiotensin converting enzyme inhibitor (ACEI) or Angiotensin II receptor blockers (ARBs) treatment as recommended by ADA. Some had deranged renal function that is a contraindication for the use of ACEI/ARBs usage. Further analysis of our study revealed that hypertension and high HbA1c were important risk factors for microalbuminuria as described by previous studies. Walraven et al. have shown that raised HbA1c have direct relationship with microalbuminuria.^15^ While other studies have shown that age, serum creatinine and hypertension is important in microalbuminuria,^2^,^13^ however our study failed to show any significant association of age with microalbuminuria. Smoking, alcohol intake were also noted to be an important risk factor by other studies^13^ but in our study this difference was also insignificant.

Our study showed that chronic complications of diabetes like diabetic retinopathy and neuropathy and ischemic heart disease were also more common in microalbuminuric group as compared to non microalbuminuric group and literature showed that prompt treatment reduces these complications.^16^

Patients with microalbuminuria had significantly more dyslipidemia and the major significant difference was triglycerides level. HDL and LDL fail to show any significant difference between albuminuric and non albuminuric groups. It can be explained by the fact that uncontrolled diabetes is not only a causative factor for microalbuminuria but it also increases the triglyceride levels. However our study showed that difference between hypertensive vs. non hypertensive patients is LDL cholesterol. As major risk factor for macrovascular disease in diabetics is dyslipidemia^17^ this signifies that these patients are at increased risk of cardiovascular events. Our study also highlight the higher usage of statin therapy among the albuminuric patients but the risk reduction benefits of lipid lowering therapy in Asian population is not well established.^18^

Our study also revealed that there is increased usage of insulin in microalbuminuric group as compare to those who have no microalbuminuria, it signifies that their diabetes were difficult to control on oral hypoglycemics only.

## CONCLUSION

The frequency of microalbuminuria in our study is 31.56% and it is strongly associated with hypertension, poor glycemic control and other diabetic complication like neuropathy and retinopathy. Although it is in accordance to other areas of world but as the burden of diabetes is too high in our country, it forecast a huge number of patients with renal and other diabetic complications in the near future and necessitates immediate intervention to stop this future havoc.

### Authors` Contribution

**TA** conceived and designed study, did literature search, collected and analyzed data.

**IU** drafted initial manuscript and helped in literature search.

**MM** helped in analysis and drafting final manuscript.

**NI** contributed in supervising the study.
